# Association between type 1 diabetes mellitus and ankylosing spondylitis: a two-sample Mendelian randomization study

**DOI:** 10.3389/fimmu.2023.1289104

**Published:** 2023-12-20

**Authors:** Ju Zhang, Jiaping Qi, Yixuan Li, Jing Wang, Huan Jiang, Qiong Sun, Qinchen Gu, Zhenhua Ying

**Affiliations:** ^1^ Jinzhou Medical University Graduate Training Base Zhejiang Provincial People's Hospital, Center for General Practice Medicine, Department of Rheumatology and Immunology, Zhejiang Provincial People's Hospital, Hangzhou, Zhejiang, China; ^2^ Zhejiang Provincial Key Laboratory of Traditional Chinese Medicine Cultivation for Arthritis Diagnosis and Treatment, Zhejiang Provincial People's Hospital, Affiliated People's Hospital, Hang zhou Medical College, Hangzhou, Zhejiang, China

**Keywords:** type 1 diabetes, ankylosing spondylitis, Mendelian randomization, causality, risk

## Abstract

**Objective:**

The development of ankylosing spondylitis (AS) is closely related to autoimmune system dysfunction. Type 1 diabetes mellitus (T1DM) is an autoimmune disease that is a risk factor for many diseases. This study aimed to investigate the causal relationship between T1DM mellitus and AS genetically.

**Methods:**

A genome-wide association study (GWAS) of causal relationships between exposure (T1DM) and outcome (AS) was performed using summary data from the GWAS database. We conducted a two-sample Mendelian randomization (MR) study of these two diseases. Inverse variance weighting (IVW) was used as the primary analysis method, with MR Egger, weighted median, and weighted mode used as supplementary methods. Sensitivity analyses were performed using Cochran’s Q test, MR-Egger intercept, MR-Pleiotropy RESidual Sum and outlier methods, leave-one-out analysis, and funnel plots.

**Results:**

A total of 11 single nucleotide polymorphisms (SNPs)were identified for instrumental variables(IVs) for MR analysis.IVW found that T1DM was causally associated with AS ((IVW: OR = 1.0006 (95% CI 1.0001, 1.0011), p = 0.0057; MR-Egger: OR = 1.0003 (95% CI 0.9995, 1.0012), p = 0.4147; weighted median: OR = 1.0006 (95% CI 1.0003, 1.0008), p = 0.0001; weighted mode: OR = 1.0007 (95% CI 1.0005, 1.0009), p = 0.0001). No horizontal pleiotropy was found for the MR-Egger intercept, and leave -one-out analysis found that the results remained stable after the removal of individual SNPs.

**Conclusion:**

The results of the two-sample MR analysis supported a causal relationship between T1DM and AS risk.

## 1 Introduction

Ankylosing spondylitis (AS) is a common inflammatory arthritis that mainly involves the sacroiliac and spinal joints, and progresses gradually from the lower back to the cervical region, with clinical manifestations of lower back pain, morning stiffness, arthritis, and attachment-point inflammation. AS is a chronic autoimmune inflammatory disease of unknown etiology (1). The intercept AS incidence is about approximately 0.1-0.9% globally (2), with a 2-3-fold higher in incidence in males than females (3). It has a high teratogenicity rate and, except for rheumatoid arthritis, is the leading disability. AS has a marked familial aggregation and genetic correlation, with a 30% probability of being inherited by a relative of a descendant; the age of onset tends to be progressively younger, and AS has become a major economic burden for the healthcare systems of various countries (4, 5). AS is a disease of complicated etiology, with genetic predisposition, and environmental factors, and inflammatory cell activation can all contribute to its development (6).Recently, with continued advances in diagnosis and treatment.AS survival rates have improved dramatically, however the morbidity rates have not improved significantly. Therefore it is essential to continue to identify AS risk factors.

Type 1 diabetes mellitus (T1DM) manifests as the destruction of pancreatic islet function leading to an absolute lack of insulin secretion and production of specific insulin autoantibodies (7). The disease occurs predominantly in adolescents and manifests as a hypermetabolic syndrome with excessive thirst, polydipsia, polyuria, and elevated blood glucose. Approximately 50% of patients develop the disease in adulthood (8), and nearly half of adults with T1DM are misdiagnosed with type 2 diabetes.T1DM is an autoimmune disease in which reactive T-lymphocytes attack β-cells, causing them to become dysfunctional and mediating the production of a wide range of auto-antibodies directed against insulin (9, 10). When certain danger signals stimulate pancreatic β-cells, immune cells accumulate and activate specific insulin autoantibodies. When immune cells are aggregated, T lymphocytes are activated, increasing infiltration of naïve reactive T cells, producing INFa, and triggering NF-κB activation in β-cells (9). Diabetes mellitus is a risk factor for various of diseases, and studies have shown interconnections with rheumatoid arthritis, AS, osteoporosis, and systemic lupus erythematosus(SLE) (11–13). Diabetes mellitus is a major risk factor for several diseases. T1DM incidence has increased significantly worldwide over the past few decades, and researchers have made more progress in studying the comorbidities of T1DM with various autoimmune disorders (14). A recent Mendelian randomization (MR) study analyzed the association between T1DM onset and altered intestinal microflora (15). Another MR study found a genetic causality between T1DM and SLE (16). Previous observational studies have shown that diabetes mellitus is associated with AS (13). However, a causal relationship between T1DM and AS remains unclear.In this study, we investigated whether a causal relationship exists between T1DM and AS using MR analysis.

Epidemiological studies have shown strong associations between various risk factors and diseases, Causality in observational studies is susceptible to interference from potential confounders and reverse causality. MR is an analytical method used in epidemiological studies to assess genetic causality between exposure and outcome through genetic variation that occurs during base pairing, with single nucleotide polymorphisms (SNPs) serving as instrumental variables (IVs) (17). MR analyses are can avoid the effects of confounding factors and the reverse causality, associated with traditional epidemiological methods (18). MR analyses have been widely used.

## 2 Materials and methods

### 2.1 Data sources and research design

Single-nucleotide polymorphisms (SNPs) from the genome-wide association study (GWAS) database as IVs (19). Significant SNPs (P<5×10^-8^) associated with the associated T1DM were obtained from the FinnGen database and included 185,115 people of European ancestry (2,542 cases and 182,573 controls), while significant SNPs associated with AS (P<5× 10^-8^) were obtained from the GWAS database including 337,159 people of European origin (968 cases and 336,191 controls) (**Table 1**).

**Table 1:** Details of the GWAS included in the Mendelian randomization.

Year	Trait	Population	Case	Controls	Samplesize	Web sourse
2021	Type1 diabetes	European	2,542	182,573	185,115	www.finng,fi/en
2017	Ankylosing spondylitis	European	968	336,191	337,159	http://gwas.mrcieu.ac

**Figure 1:**
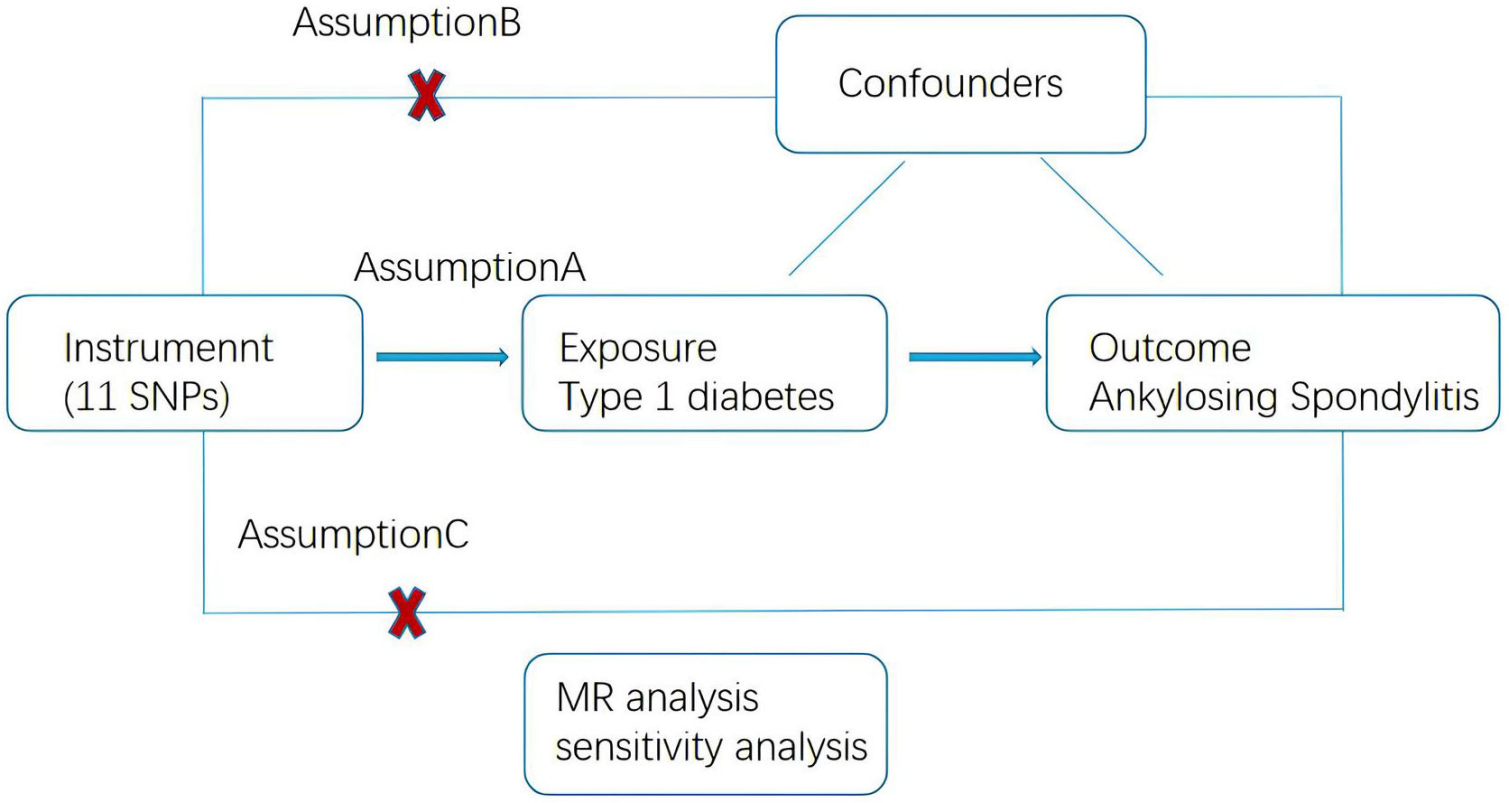
Schematic of the Mendelian randomization framework. The three core assumptions were as follows: **(A)** the single nucleotide polymorphisms (SNPs) should be strongly as associated with type 1 diabetes. **(B)** the SNPs should not be related to confounders, and **(C)** the SNPs should not be directly associated with ankylosing spondylitis.

A two-sample MR approach was used to investigate the and potential causal relationship between T1DM and AS. This MR study was conducted in accordance with the Strengthening the Reporting of Observational Studies in Epidemiology using MR (STROBE-MR) statement (20). The study design is illustrated in **Figure 1**. The MR approach should fulfill three assumptions: first, genetic variance should be associated with T1DM; second, genetic variance should not be associated with any confounding factors, and third, genetic variants affect on AS only through T1DM.

To avoid the potential bias caused by strong linkage disequilibrium (LD), we selected SNPs with LDr^2^ < 0.001. Eleven SNPs were chosen as the IVs. In addition, to avoid weak IV bias, we calculated the F for each IV-SNP using the following formula: F = R^2^ × (N -2)/(1 - R^2^),R^2^ = 2×EAF×(1−EAF)×β^2^ statistic: where R^2^ is the genetic variance explained by each SNP and N is the sample size of the exposed dataset, EAF refers to the effect allele frequency and β refers to the estimated effect of SNP,IVs with F-statistics lower than 10 were considered to have a weaker potential to predict AS and were excluded.

### 2.2 Statistical analysis

**Figure 2:**
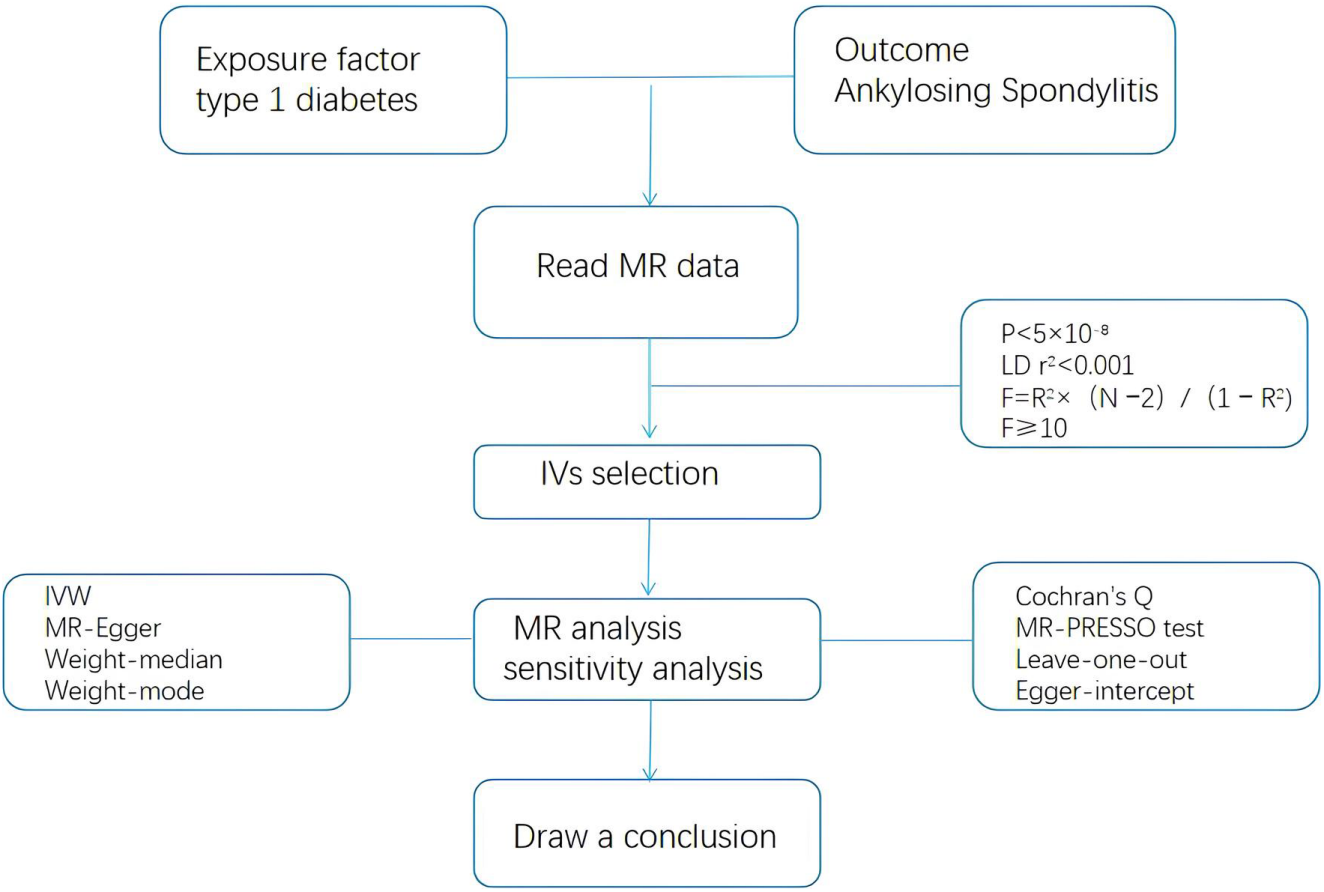
Flow chart of Mendelian randomization analysis.

The statistical analysis process is illustrated in **Figure 2**. The selected SNPs and AS data were entered into the R language package (version 4.2.1) for MR analysis. The selection of IVs meets the above criteria (P < 5 × 10^−8^, LDr^2^<0.001, F=R^2^× (N −2)/(1 − R^2^), F>10). Random effects inverse variance weighting (IVW) was used as the main analytical method (21), and the MR Egger, weighted median,weighted models were used as supplementary methods (22).

### 2.3 Sensitivity analysis

Sensitivity analyses, including tests for heterogeneity and horizontal pleiotropy, were vital in determining whether the results of the MR analyses were reliable. Cochran’s Q test was used to assess the heterogeneity of effect sizes of the selected genetic IVs, with a p-value of the Q test < 0.05 or I2 > 75% indicating the presence of heterogeneity. If heterogeneity was present, a random-effects IVW model was used (23). The MR-Pleiotropy RESidual Sum and Outlier approach (MR-PRESSO) was performed to exclude outliers and horizontal polytomies (24). Vertical polytomies were assessed using intercepts derived from MR-Egger regressions (23). We performed leave-one-out analyses to explore the effect of removing of selected individual SNPs on the overall results (25).

## 3 Results

### 3.1 Results of instrumental variables selection

We extracted 11 SNPS (P<5×10^−8^) F statistics >10 were significantly associated with TIDM from GWAS, indicating no weak instrumental bias.

### 3.2 Results of MR analysis

**Figure 3:**
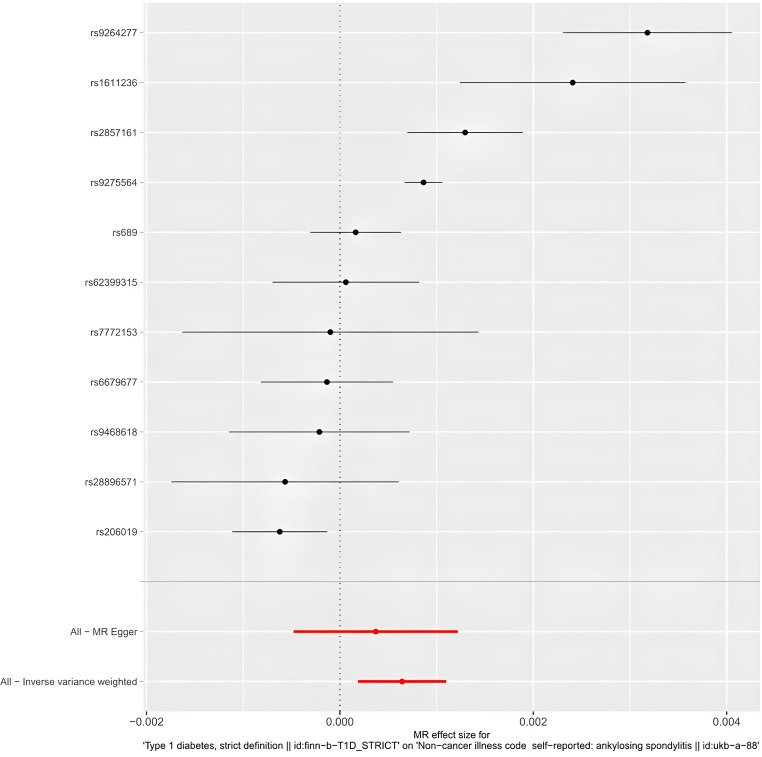
The forest plot of Mendelian randomization analysis for 11 single nucleotide polymorphisms.

**Figure 4:**
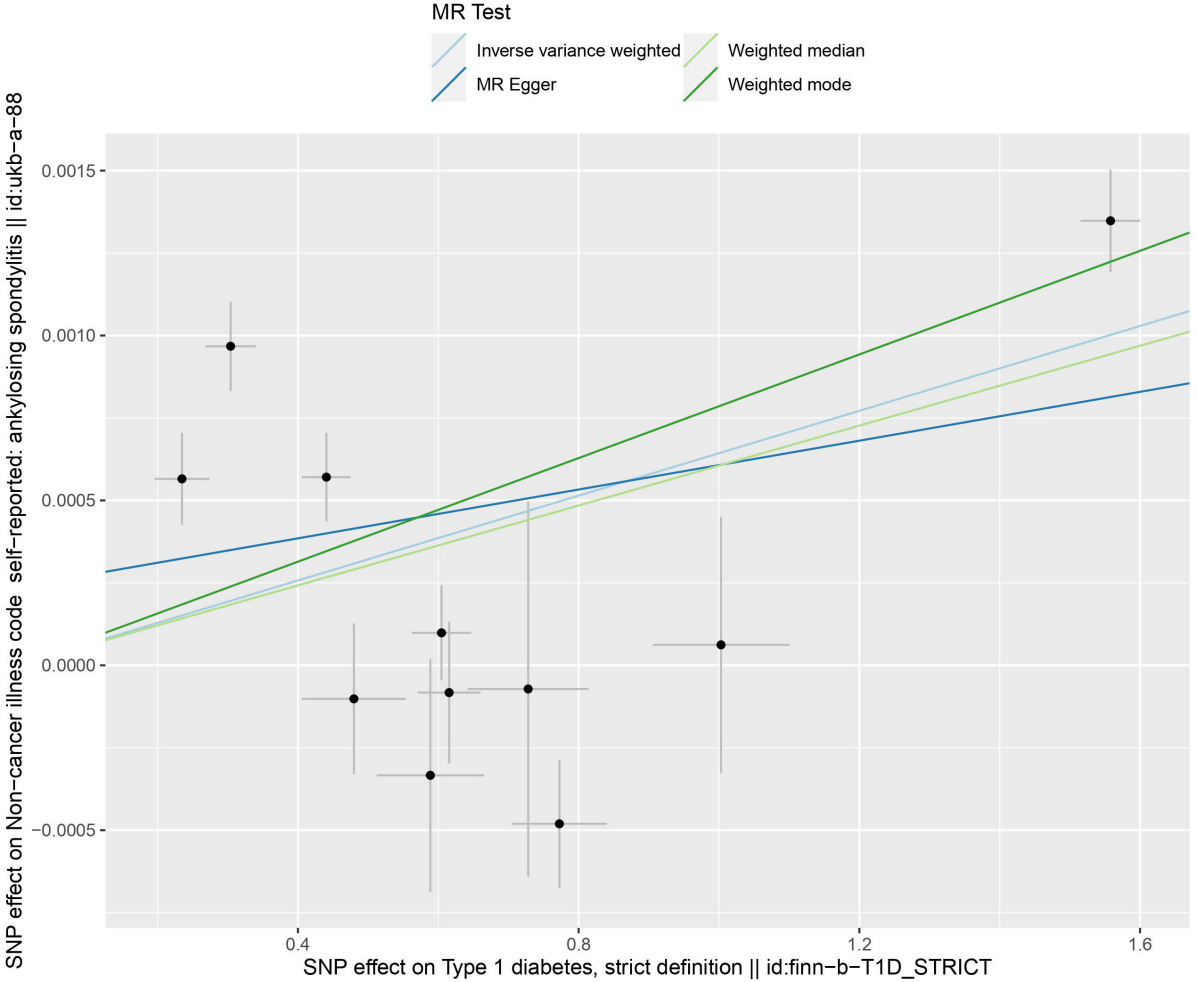
The scatter plot of four Mendelian randomization methods.

IVW results indicate a genetically determined association between T1DM and AS (IVW: OR = 1.0006 (95% CI 1.0001, 1.0011), P= 0.0057; MR-Egger: OR = 1.0003 (95% CI 0.9995, 1.0012), P = 0.4147; weighted median: OR = 1.0006 (95% CI 1.0003, 1.0008), P= 0.0001; weighted mode: OR = 1.0007 (95% CI 1.0005, 1.0009), P= 0.0001). A causal relationship between was observed T1DM and AS (**Table 2**) (**Figures 3**, **4**).

**Table 2:** MR results and sensitivity analysis for association of T1DM and AS risk.

Method	nSNP	OR	95%CI	SE	P-value	Q(P-value)	Intercept (P-value)
Inverse variance weight	11	1.0006	1.0000–1.0011	0.0002	0.0057	<0.0001	
MR-egger	11	1.0003	0.9995–1.0012	0.0004	0.4147	<0.0001	0.4698
Weight medlian	11	1.0006	1.0003–1,0008	0.0001	0.0001		
Weight mode	11	1.0007	1.0005–1.0009	0.0001	0.0001		

### 3.3 Results of sensitivity analysis

**Figure 5:**
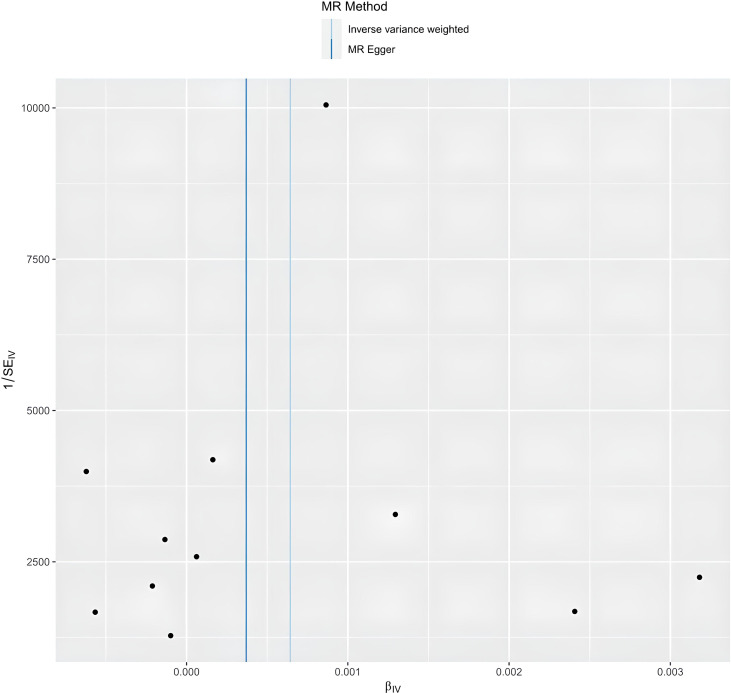
The funnel plot of Mendelian randomization analysis.

**Figure 6:**
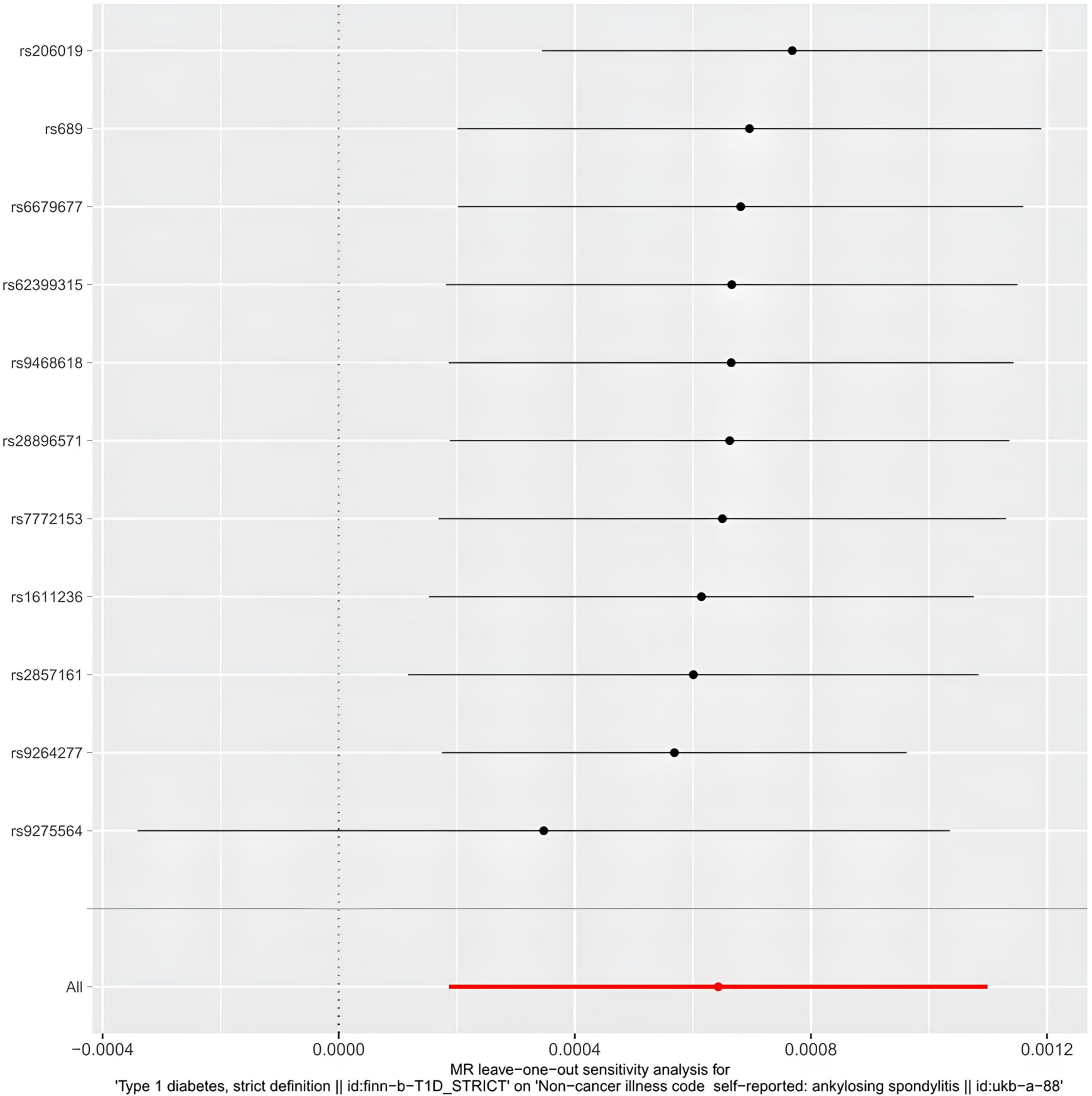
The forest plot of leave-one-out analysis.

The egger intercept(P= 0.4698) in the MR analysis and the funnel plot showed no horizontal pleiotropy (**Figure 5**). Heterogeneity tests showed heterogeneity between SNPs (MR-Egger: Q = 90.04, P < 0.0001, IVW: Q = 95.73, P < 0.0001) Since the two samples had data from different populations, the random-effects IVW approach allowed for heterogeneity arising from SNPs. The leave-one-out analysis revealed that the results were stable and not affected by single SNPs (**Figure 6**).

## 4 Discussion

MR analysis based on pooled GWAS data explored the genetic between exposure (T1DM) and outcome (AS) using from four methods (IVW, weighted median, weighted mode, and MR-Egger) and concluded that there was evidence of a causal relationship between T1DM and AS.

Research analyses and case reports have revealed a correlation between T1DM and rheumatic diseases (16, 26). In the present MR analysis, a positive causality of T1DM on the risk of AS was observed. T1DM is often comorbid with multiple autoimmune diseases, with autoimmune thyroid disease being common, in addition to celiac disease, skin disorders, and connective tissue disorders not being uncommon (26). Recently, researchers have investigated the relationship between insulin deficiency and rheumatic diseases. A single-center cross-sectional study showed that insulin resistance was evident during early arthritis development, whereas disease activity and age were independent predictors of insulin resistance (27). Case reports have also confirmed increased levels of anti-insulin antibodies in patients with SLE (16). Currently, there are fewer studies on the relationship between T1DM and AS, and the lack of sample sizes supports the elimination of confounding bias, as well as an indication of causality. Causality was also considered

Our study found a direct genetic causal relationship between T1DM and AS. There are several possible explanations for the increased AS risk in patients with T1DM. First, T1DM is an autoimmune disease and immune cells dysfunction may play a key role in AS pathogenesis. Several studies have confirmed that B-cell dysfunction and reactive autoantibody production in patients with AS are important in the development of the disease (28). In T1DM, B-cells produce insulin antibodies, which stimulate T-lymphocyte activation and value-addition by presenting autoantigens through insulin antibodies and mediate autoimmune cytotoxicity inducing AS (29). B-cells have been shown to increase the risk of AS in patients with T1DM; however, the risk is not as high as that in patients with T1DM. In addition, hyperglycemia disrupts inflammatory cells and T and B cell immune functions in patients with autoimmune diseases. Y et al. reported that hyperglycemia induces upregulation of CD4+ T cell glycolysis and mitochondrial oxidative metabolism, leading to elevated levels of immune response (30). Second, genetic susceptibility plays a vital role in the induction of AS by T1DM. Numerous studies have proposed that the presence of autoimmune diseases in combination may be associated with the selective binding of two discrete epitopes of the same autoantigen to different human leukocyte antigens (HLAs) to produce two specific antibodies that induce disease production. In addition, autoantigens may bind to either HLA proteins resulting in the induction of both diseases through cross-presentation (31, 32). Later, investigators also identified motifs sharing immune-mediated functions within the HLA region, as well as outside the region in autoimmune diseases, including cytotoxic T-lymphocyte antigens 4 and tumor necrosis factor(TNF)-inducible proteins 3, which have been implicated in genes whose phenotypes are associated with B or T cell activation and differentiation, innate immunity, cytokines, innate immunity and cytokine signaling regulation related pathways associated with the development of specific autoimmune diseases (33). Third, environmental factors contribute to disease development. Obesity, infections (especially viral infections), intestinal flora dysbiosis (especially bifidobacteria), and dietary behaviors are closely associated with the development of T1DM (34), and these risk factors have been shown to contribute to the development of rheumatic disorders (35, 36). Significant intestinal dysbiosis is observed in patients with diabetes (37). Studies have confirmed that intestinal flora disorders are closely associated with the development of AS (38). In animal experiments, modulation of the intestinal flora alleviated the inflammatory response of AS in mice (39). Although recent two-sample MR analyses have demonstrated no causal relationship between intestinal flora dysbiosis and the development of AS (40), intestinal flora dysbiosis is a risk factor for AS. Finally, evidence for pharmacotherapy comes from the therapeutic use of the hypoglycemic drug metformin in rheumatic diseases. Metformin is globally recognized as a first-line hypoglycemic agent that not only lowers glucose levels, but also exerts anti-inflammatory and immunomodulatory effects (41). Its ability to reduce the production of neutrophil extracellular traps by inhibiting the nicotinamide adenine dinucleotide phosphate oxidation pathway and reducing mitochondrial DNA-induced TNF-a effectively reduces inflammatory responses in SLE (42).Secondly, metformin also reduces the production of TNF-a by inhibiting the AMP-activated protein kinase pathway and reduces fibroblast osteoclastogenesis, reducing the inflammatory responses, which is effective effect in the treatment of AS (43). In addition, hydroxychloroquine and TNF-a inhibitors can improve glucose metabolism and reduce the risk of diabetes mellitus in patients with AS (44). In conclusion, T1DM and AS are complex autoimmune inflammatory diseases, and inflammation is the central part of the disease response, with the body’s immune system becoming dysfunctional and the inflammatory pathway being activated. The dysfunction and activation of inflammatory pathways may be the most critical factors in the development of AS induced by T1DM.

### 4.1 Strengths

This study has a number of strengths. First, to the best of our knowledge, this is the first study to propose a causal relationship between T1DM and AS, and our findings provide a reference for the development of both diseases. Second, the MR approach avoids confounding factors in observational studies and the large-scale GWAS dataset makes the statistical power more adequate to detect causality.

### 4.2 Limitations

This study has some limitations. First, most of the samples included in the GWAS GeneBank are European, with only a small Asian populations, therefore, the MR analysis was performed in European populations, and the results do not extrapolate well to non-European populations. Second, we chose an overall small sample size, with a weak causal relationship between the two samples, and the positive results were not significant enough to be supported by a larger sample. Third, Third, our study emphasized the effect of T1DM on AS. Because of the limited available SNPs associated with AS showing low F-statistics (<10) (49), we did not perform a bidirectional MR Analysis. We could not provide a reasonable explanation for the causal relationship between AS and T1DM because of the challenges of the existing GWAS data and the lack of stable IVs. Finally, this study provided only genetic evidence and did not address environmental factors.

## 5 Conclusion

The results of the MR study support a causal relationship between T1DM and AS, and this MR study is the first to investigate the causal relationship between T1DM and AS, reducing bias in observational studies. The mechanisms underlying this association require further investigation. However, we cannot ignore the higher risk of AS due to risk factors such as inflammatory burden, genetic susceptibility, and the environment in patients with T1DM. Early management of blood glucose leaves is necessary in patients with AS.

## Data availability statement

The datasets presented in this study can be found in online repositories. The names of the repository/repositories and accession number(s) can be found in the article/supplementary material.

## Author contributions

JZ: Writing – original draft. JQ: Writing – original draft. YL: Data curation, Methodology, Writing – review & editing. JW: Data curation, Methodology, Writing – review & editing. HJ: Data curation, Writing – review & editing, Formal Analysis. QG: Writing – review & editing, Data curation. QS: Writing – review & editing, Methodology. ZY: Writing – review & editing, Funding acquisition.
